# Carnosine Dipeptidase(Cndp): An emerging therapeutic target for metabolic diseases and cancers

**DOI:** 10.1016/j.gendis.2025.101804

**Published:** 2025-08-13

**Authors:** Liang Zhou, Shuxia Zhang, Yunqi Zhang, Yun Luo, Xiaobo Sun

**Affiliations:** a State Key Laboratory for Quality Ensurance and Sustainable Use of Dao-di Herbs, Institute of Medicinal Plant Development, Peking Union Medical College and Chinese Academy of Medical Sciences, Beijing 100193, China; b Beijing Key Laboratory of Neuro-Innovative Drug Research and Development of Traditional Chinese Medicine (Natural Medicines), Beijing 100193, China; c Key Laboratory of Bioactive Substances and Resource Utilization of Chinese Herbal Medicine, Ministry of Education, Beijing 100193, China; d Academy of Chinese Medicine Sciences, Henan University of Chinese Medicine, Zhengzhou, Henan 450046, China

**Keywords:** CNDP1, CNDP2, Diabetic nephropathy, Metabolic diseases, Therapeutic target

## Abstract

Metabolic diseases, associated with high morbidity and mortality rates, pose a challenge to global public health and a significant burden on society. Since the discovery of carnosinase-2(CNDP2)-mediated synthesis of lactate and phenylalanine, which subsequently forms N-Lactoyl-Phenylalanine (Lac-Phe) to inhibit food intake and obesity, the carnosine dipeptidases (CNDPs)have attracted increasing scientific interest. Although the role of CNDP in diabetic nephropathy has been extensively studied, its role in other metabolic diseases remains unclear. In this study, we have overviewed the enzymatic and other roles of CNDP proteins focusing on recent research demonstrating the regulatory roles of CNDP on various metabolic diseases. Increasing evidence indicates that carnosinase-1(CNDP1) and carnosinase-2 are crucial for the management of metabolic diseases under both physiological and pathological conditions. Moreover, interest in the pharmacological modulators of CNDP has been steadily increasing. Overall, we suggest that CNDP can be considered a promising therapeutic target for the effective treatment of metabolic diseases.

## Introduction

Carnosine dipeptidase (CNDP) enzymes are classified into two types: carnosinase-1 (CNDP1; also known as carnosine dipeptidase 1, serum carnosinase, and EC 3.4.13.20) and carnosinase-2 (CNDP2; also known as carnosine dipeptidase 2, cytosolic non-specific dipeptidase, and EC 3.4.13.18).^1^ Cytosolic non-specific dipeptidase 1 and its homologous protein CNDP2 are dipeptide metalloproteinases belonging to the M20 family.^2^

CNDP1 is encoded by the CNDP1 gene, situated on the long arm of chromosome 18 (18q22.3). This gene spans approximately 50.6 kb and is comprised of 12 exons. CNDP2 is located on chromosome 18, is 13 kb telomeric from CNDP1, and spans approximately 24.8 kb with 12 exons. Both genes are arranged in a tail-to-head orientation on the chromosome.^3^

CNDP1 and CNDP2 are encoded by the carnosinase genes and are pivotal to the metabolism of carnosine and related dipeptides. CNDP hydrolyzes carnosine and functions as a non-specific enzyme that synthesizes dipeptides. Under appropriate conditions, human isoforms of carnosinase (CNDP1 and CNDP2) catalyze the hydrolysis of dipeptides, such as carnosine (β-alanyl-l-histidine) and homocarnosine (γ-aminobutyryl-l-histidine).^4^

The difference is that CNDP1 is an enzyme that specifically degrades carnosine (β-alanyl-l-histidine) and anserine (β-alanyl-N-methylhistidine), two natural histidine-containing dipeptides. It belongs to the M20 metalloprotease family and comprises a catalytic domain with a dinuclear Zn^2+^ center; moreover, it exhibits a narrow substrate specificity.^5^ CNDP1 is involved in carnosine metabolism, and carnosine homeostasis plays a role in various physiological functions.^6^ The CNDP2 gene encodes a non-specific carnosine peptidase with a high affinity for Cys–Gly in the γ-glutamate cycle and is involved in glutathione biosynthesis.^7^ Moreover, CNDP2 mediates the reverse proteolysis of lactate and amino acids.^8^

Additionally, CNDP1 and CNDP2 exhibit therapeutic effects against various diseases. They are involved in various physiological processes. For example, CNDP1 has a potential protective role against the long-term consequences of reactive metabolite accumulation in conditions such as diabetes mellitus. The accumulation of carnosine and anserine in the kidneys owing to CNDP1 knockout suggests a potential therapeutic strategy for alleviating or preventing chronic kidney diseases, including diabetic nephropathy.The key molecular and biochemical features of CNDP1/2 are illustrated in Table 1.

**Table 1:** Summary of the basic molecular and biochemical properties of CNDP1/2.

	Carnosinase-1	Carnosinase-2
Structural formula and substrate	(PDB-3dlj)	(PDB-4ruh)
Aliases	CNDP1, carnosine dipeptidase 1, serum carnosinase, and EC 3.4.13.20	CNDP2, carnosine dipeptidase 2, cytosolic non-specific dipeptidase, and EC 3.4.13.18
Gene structure		
Physiological functions	≈50.6 kb (∼18 kb ≈ 12 exons); specific peptidase; M20 metalloproteinase family; Zn^2+^ dinuclear catalytic center; high substrate specificity	≈24.8 kb (∼12 kb ≈ 12 exons); non-specific peptidase; participates in the γ-glutamyl cycle; hydrolyzes Cys–Gly and participates in glutathione biosynthesis
Role in disease	Related to diabetic nephropathy and oxidative stress metabolism	Participates in metabolic regulation potential anti-disease effect

In summary, CNDP1 and CNDP2 are crucial genes that encode carnosinase enzymes involved in the degradation of carnosine and related dipeptides. CNDP1 and CNDP2 play a pivotal role in determining susceptibility to various metabolic disorders like diabetic nephropathy; hence, these are potentially significant targets for ongoing research and potential therapeutic interventions. This article reviews the significant roles of the CNDP family of proteins in their functions in normal physiology and diseases (metabolic diseases and cancer).

## Physiological functions of CNDP

The CNDP gene-encoded enzymes include the human carnosinase isozymes CNDP1 and CNDP2. CNDP1, encoded by *CNDP1*, is a zinc-dependent and substrate-specific enzyme. It plays a crucial role in the degradation of carnosine and anserine *in vitro* and *in vivo*.^5^^,^^9^ *CNDP2*-encoded CNDP2 possesses similar dipeptidase activity; however, its substrate specificity varies in the presence of different metal ions, including zinc and manganese.^10^ Furthermore, CNDP2 in drosophila models was reported to exhibit non-specific dipeptidase activity in the cytoplasm.^11^

As enzymes, CNDP1 and CNDP2 share similarities, catalyzing the hydrolysis of dipeptides, such as carnosine (β-alanyl-l-histidine) and homo carnosine (γ-aminobutyryl-l-histidine),^4^ and also display similar clinical manifestations. CNDP1 and CNDP2, serving as enzymes, exhibit their diversity and significance in carnosine metabolism, dipeptidase activity, and overall cellular metabolism.

In addition, CNDP2 has many different effects. CNDP2 can hydrolyze proteins in either a forward or reverse manner. CNDP2, a cytosolic enzyme, hydrolyzes carnosine to produce l-histidine and β-alanine. The discovery that N-lactoyl amino acids are universal metabolites produced by the CNDP2-mediated reverse proteolysis of lactate and amino acids has expanded our understanding of their function.^8^ Furthermore, electrospray ionization mass spectrometry studies indicate that the interaction between the H228 residue and the reaction center of the dimeric counterpart plays a key role in the catalytic function of CNDP2.^12^

CNDP2 significantly impacts intracellular amino acid and dipeptide metabolism. *In vivo* metabolomic analyses fully annotated the threonyl dipeptide metabolic pathway regulated by CNDP2.^13^ CNDP2 protects macrophages from cysteine deficiency by hydrolyzing glutathione-related peptides.^14^ However, in human proximal tubular cells, CNDP2 knockout affects dipeptide and amino acid homeostasis, disrupting both intracellular and transcellular solute transport.^15^ This fully demonstrates that CNDP2 plays an active role in intracellular amino acid and dipeptide metabolism.

Among the catalytic roles played by CNDP2, it is noteworthy that Lac-Phe is synthesized from lactate and phenylalanine in CNDP2^+^ cells, including macrophages, monocytes, and various immune and epithelial cells distributed across different organs.^16^ Exercise induces the production of N-lactylphenylalanine (Lac-Phe), a signaling metabolite in the blood, which suppresses appetite and decreases obesity. The regulatory role played by CNDP2 is not limited to this. Intestinal epithelial CNDP2^+^ cells rather than macrophages are the primary source of both basal and metformin-induced Lac-Phe *in vivo*. The CNDP2/Lac-Phe pathway acts as a critical mediator of the effects of metformin on energy balance.^17^^,^^18^ The expression of CNDP2 is also affected by other factors. In mice and rats, CNDP2 expression was up-regulated in the adipose tissue after t10-c12 CLA treatment and caloric restriction, respectively; this result indicates a potential isoform-specific effect of t10-c12 CLA on CNDP2 expression. Another report proposed that the t10-c12 CLA treatment- and caloric restriction-associated increase in CNDP2 expression may enhance its anti-obesity properties, which could be attributed to elevated Lac-Phe levels; these changes may reduce daily food intake, body weight, and fat mass.^19^

One of the reasons why CNDP2 has been studied more deeply and applied more widely than CNDP1 may be the widespread distribution of CNDP2 mRNA. The purification and identification of CNDP2 in Japanese eels further confirmed its conservation and significance across different organisms; the mRNAs of CNDP1 and Xaa-methyl-His dipeptidase (or anserinase: EC 3.4.13.5, ANSN) were more abundant in the liver than in other tissues of the Japanese eel, whereas CNDP2 mRNA was widely distributed across all tested tissues.^20^ There are many studies on the mRNA level of CNDP2. For example, another report demonstrated higher levels of CNDP2 mRNA and the carnosine transporters in oxidative muscle fibers than in glycolytic muscle fibers, suggesting a potential role of this transporter in carnosine uptake and/or efflux and maintenance of cellular homeostasis.^21^

The second reason why CNDP2 has been studied more deeply and applied more widely than CNDP1 may be that CNDP2 can not only catalyze as an enzyme alone, but also work synergistically with other substances. For example, CNDP2 collaborates with aldehyde dehydrogenase 1 family member A1 (ALDH1A1) and nicotinamide nucleotide adenylyltransferase 1 (NMNAT1) to play a key role in reducing protein oxidation.^22^ To understand the role of carnosine and CNDP2 in the brain, researchers explored the immunohistochemical localization of CNDP2 in the hypothalamus and demonstrated high expression of CNDP2 in histaminergic neurons of the tuberomammillary nucleus; this suggests that CNDP2 potentially supplies histidine to these neurons for histamine synthesis.^23^ This suggests that CNDP2 may play an important role in neurons. It is worth mentioning that CNDP1 plays a unique role in regulating development-related characteristics, further underscoring its functional diversity across various tissues and developmental stages.^24^ In summary, CNDP2 plays a significant role in reaction catalysis, proteolysis, and amino acid and dipeptide metabolism, and has the characteristics of wide distribution and synergistic reaction. CNDP1 and CNDP2, especially CNDP2, exhibit multiple regulatory functions.

In summary, the CNDP proteins play indispensable roles in several physiological processes, including carnosine metabolism, dipeptidase activity, and cellular metabolism.

## Function in diseases (metabolic diseases and cancers) of CNDP

CNDP1 and CNDP2 are considered important biomarkers of obesity, metabolic diseases, and other physiological functions.

## Obesity

CNDPs (especially CNDP1 and CNDP2) are crucial for regulating metabolism and are the topic of growing interest in obesity-related research. Oxidative stress and inflammation are pivotal for the onset of chronic metabolic disorders, such as obesity. Carnosinase (CNDP1) and cell non-specific dipeptidase (CNDP2) play vital roles in protecting cells and tissues from oxidative stress. For example, trans-10, cis-12 conjugated linoleic acid and caloric restriction up-regulate CNDP2 expression in rodent white adipose tissue, affecting feeding and obesity.^19^

**Figure 1:**
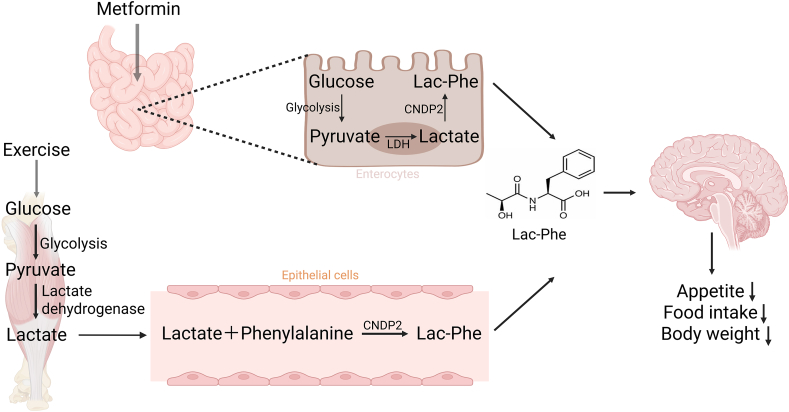
CNDP2 suppresses obesity by catalyzing the synthesis of Lac-Phe. The solid lines represent validated pathways, and the dashed lines represent hypothesized pathways.

CNDP2 catalyzes the biosynthesis of N-lactoyl-phenylalanine (Lac-Phe) from lactate and phenylalanine. Lac-Phe is a molecular effector associated with physical activity in various mammalian species. It selectively suppresses food intake and obesity in mice fed a high-fat diet, and functions as a conserved exercise-induced metabolite that regulates food intake and influences whole-body energy balance.^16^ Figure 1 illustrates the process of Lac-Phe production and its impact on appetite and energy homeostasis.

Additionally, intestinal epithelial CNDP2^+^ cells rather than macrophages were the primary source of both basal- and metformin-induced Lac-Phe *in vivo*. Lac-Phe critically mediates the body weight-lowering effects of metformin.^17^^,^^18^ Metformin, a potent pharmacological inducer of Lac-Phe, stimulates Lac-Phe biosynthesis by inhibiting complex I, enhancing glycolytic flux, and promoting the mass production of intracellular lactate. Genetic ablation of Lac-Phe biosynthesis in male mice induces resistance to the effects of metformin on food intake and body weight.

Moreover, single-nucleotide polymorphisms in carnosinase genes (CNDP1 and CNDP2) are associated with the strength exercise status. This reflects a unique impact of the CNDP genotype on exercise development, which potentially influences the occurrence of obesity.^25^ Another report found that a combination of genetic variations in CNDP1 and CNDP2, along with dietary carotene and/or carbohydrate, influenced differences in body mass index and obesity risk between individuals. Schmöhl et al demonstrated altered amino acid metabolism and inhibited weight gain by knocking out CNDP1 in zebrafish.^26^ CNDP1 may become a therapeutic target for the treatment of metabolic syndrome, leading to effective management of obesity and associated comorbidities.^27^ In particular, the interaction between the CNDP2 genotype and dietary carotene/carbohydrate ratio is crucial for determining obesity risk.^28^

Therefore, CNDP1 and CNDP2 play various regulatory roles in obesity and associated metabolic processes, which highlights their potential as therapeutic targets. Considering the role of CNDP2 in Lac-Phe synthesis, a deeper knowledge of the regulation of CNDP2 expression across various mammalian tissues could significantly facilitate obesity treatment.

## Diabetic nephropathy

Diabetic nephropathy is a clinical syndrome characterized by persistent microalbuminuria occurring alongside insulin-dependent or non-insulin-dependent diabetes.^29^ Diabetic nephropathy is a multifactorial disease significantly associated with both genetic susceptibility and environmental factors that regulate its progression.^30^ The precise mechanisms leading to diabetic nephropathy remain unclear; however, genetic susceptibility has been recognized as a significant factor controlling its development and progression.^31^ In metabolic diseases, muscle carnosine and serum carnosinase-1 interact to modulate low-grade chronic inflammation, glucose homeostasis, and insulin sensitivity.^32^

The CNDP family proteins, particularly CNDP1 and CNDP2, are crucial for metabolic regulation and have garnered increasing attention in studies related to metabolic diseases, including diabetic nephropathy. A previous systematic review and meta-analysis revealed a significant association between CNDP1 and diabetic nephropathy.^33^ In both diabetic and non-diabetic patients with chronic kidney disease, urinary carnosinase-1 levels are correlated to serum carnosinase-1 (CN-1) rather than the CNDP1 genotype.^34^ High carnosinase 1 (CNDP1) levels are associated with diabetic kidney disease.

Genetic factors partially determine the risk of diabetic nephropathy. A gene locus on chromosome 18q22.3-q23 has been reported to be associated with diabetic nephropathy. Polymorphisms in CNDP1 are crucially associated with both diabetic nephropathy and non-diabetic chronic kidney disease.^35^ CNDP1 gene on chromosome 18q contributes to diabetic nephropathy susceptibility.^36^ Genetic variants of CNDP1 and CNDP2 are linked to varying levels of susceptibility to diabetic nephropathy. Specific single-nucleotide polymorphisms in these genes are associated with variable risks of developing diabetic nephropathy. Detailed genotyping studies demonstrated the impact of these single-nucleotide polymorphisms on disease susceptibility.^1^

CNDP1 is associated with reduced susceptibility to diabetic nephropathy. The 5-leucine repeat (5L–5L) genotype and the 5L allele of the CNDP1 gene are less abundant in patients with diabetic nephropathy than in healthy individuals and those with diabetes without kidney disease. This suggests the potential protective role of the 5L allele against kidney disease.^37^ Approximately 40% of the population is homozygous for the 5L allele of the (CTG)n repeat polymorphism in the CNDP1 gene, which is associated with reduced susceptibility to diabetic nephropathy.^38^ Additionally, patients with diabetes homozygous for the CNDP1 5L allele show resistance to diabetic nephropathy.^39^ Furthermore, the 5/5 homozygous CNDP1 genotype is infrequent among South Asian Surinamese individuals and is correlated with reduced carnosinase activity and an increased genetic risk for diabetic nephropathy.^40^

Patients with the CNDP1 Mannheim polymorphism (homozygous for five leucine repeats) have a reduced risk of developing type II diabetic nephropathy. CNDP1 Mannheim polymorphism (homozygous for five leucine repeats) is associated with a reduced risk of developing type II diabetic nephropathy in children with chronic kidney disease and other patients.^41^ The number of leucine repeats in the leader peptide of CNDP1 is correlated with susceptibility to diabetic nephropathy. Moreover, common variants of CNDP1 and CNDP2 are involved in the susceptibility to nephropathy in patients with type 2 diabetes.^1^ hCNDP1, an allelic variant of carnosinase 1 (CNDP1), partially contributes to the susceptibility to diabetic nephropathy through altered glucose metabolism in patients with type 2 diabetes.^42^

Furthermore, the effect of CNDP1 on diabetic nephropathy varies with sex. The homozygosity for the CNDP1 (CTG)5 genotype induced protection against diabetic nephropathy in women with type 2 diabetes.^43^ In patients with type 2 diabetes, a sex-specific correlation between CNDP1 and cardiovascular mortality was reported; women with the 5-leucine repeat (5L–5L) exhibited a higher risk. No association was found between CNDP1 and all-cause mortality or changes in estimated glomerular filtration rate.^44^ The Rs12604675-A variant of CNDP1 may increase the risk of overt proteinuria in Japanese women with type 2 diabetes.^45^

Moreover, CNDP1 polymorphisms are not linked to an increased risk of chronic kidney disease resulting from tubulointerstitial nephritis. Contrastingly, CNDP1 variants influence chronic kidney disease caused by chronic glomerulonephritis. The chronic kidney disease progression rate was not affected by these polymorphisms. Elevated serum carnosinase activity in patients with chronic kidney disease may indicate a role in the pathogenesis of this disease.^46^ Carnosine acts as a protective factor against diabetic nephropathy by mitigating the harmful effects of high blood glucose levels on renal cells. In BTBR ob/ob mice, carnosine treatment improved glucose metabolism, reduced albuminuria, and ameliorated pathology. Therefore, carnosine may emerge as a new therapeutic strategy for treating or preventing diabetic nephropathy in diabetic patients.^47^ Carnosine and its derivatives can support the development of new therapeutic strategies for optimized kidney protection in diabetes.^48^

CNDP1 also serves as a biomarker for diabetic nephropathy. BTBR Ob/Ob mice expressing human CNDP1 exhibit a more severe diabetic kidney disease phenotype.^49^ Consequently, CNDP1 polymorphisms predict mortality and progression from nephropathy to end-stage renal disease in diabetic patients.^50^ Moreover, CNDP1 may potentially serve as a biomarker supporting the early identification of the risk for gestational diabetes mellitus.^51^ In patients with type 2 diabetes, CNDP1 is differentially expressed in maternal plasma during the first trimester, distinguishing between pregnant women at risk for gestational diabetes mellitus and those without complications.

The potential of CNDP1 and CNDP2 as therapeutic targets has been focused on. In Malaysian patients with type 2 diabetes, CNDP1, nitric oxide synthase 3 (NOS3), and manganese superoxide dismutase (MnSOD) polymorphisms are considered risk factors for diabetic nephropathy.^52^ Additionally, cysteine compounds influence the dynamic behavior of CNDP1, indicating a promising treatment option for diabetes.^53^

In summary, CNDP1 can reduce the susceptibility to diabetic nephropathy through specific single-nucleotide polymorphisms, specific genotypes, allelic variants, *etc.*, and can also be used as a biomarker for diabetic nephropathy. Common variations in CNDP1 and CNDP2 play a role in the susceptibility of patients with type 2 diabetes to nephropathy. Dipeptide administration or increasing carnosine concentrations can protect diabetic mice from nephropathy. Among them, CNDP1 has been studied in depth, and CNDP2 has yet to be studied. Lac-Phe is a dipeptide catalyzed by CNDP2. The treatment of diabetic kidney with Lac-Phe remains to be studied, and multiple therapeutic concepts remain to be discovered. These findings suggest that CNDP1 and CNDP2 and their metabolites may pave the way for innovative therapeutic strategies to effectively protect the kidneys of diabetic patients.

## Tumor

**Figure 2:**
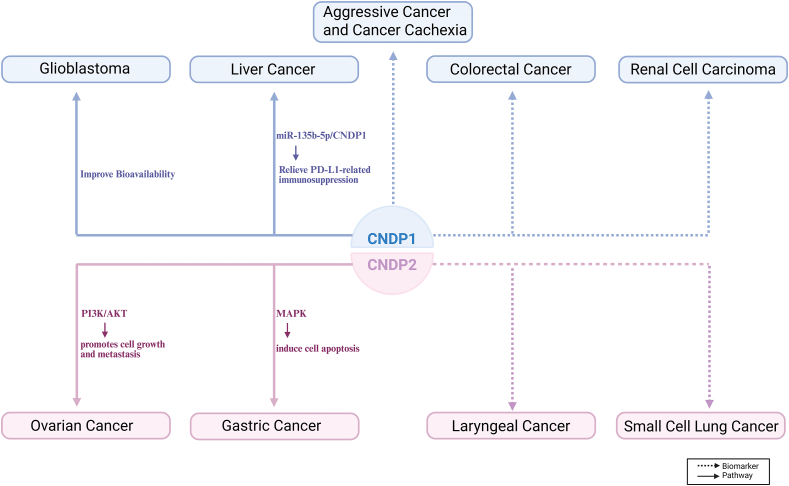
The function and potential mechanism of exchange proteins directly activated by CNDP vary across different tumors. The blue color above represents diseases where CNDP1 plays a major role, and the pink color below represents diseases where CNDP2 plays a major role.

Growing evidence indicates that CNDP2 plays several significant roles beyond its enzymatic activity and is functionally important in cancers, the leading cause of death and morbidity worldwide, which poses a serious threat to human health. Researchers predict a rapid increase in the frequency of cancer cases over the next 50 years. By 2070, the number of new cancer cases is expected to reach approximately 34 million, doubling the figure recorded in 2018.^54^ Currently, surgery, chemotherapy, and radiation therapy generally comprise the cancer treatment regimens. However, the development of chemotherapy resistance and recurrence often limits the treatment efficacy.^55^ Hence, alternative cancer treatment strategies are needed. CNDP family proteins play important roles in various tumors; previous reports indicate their potential implications as biomarkers in the diagnosis of cancers and as potential therapeutic targets. Thus, CNDP may serve as an effective target for cancer therapy. Figure 2 summarizes the potential roles and mechanisms of CNDP proteins across various types of cancers.

## Liver cancer

**Figure 3:**
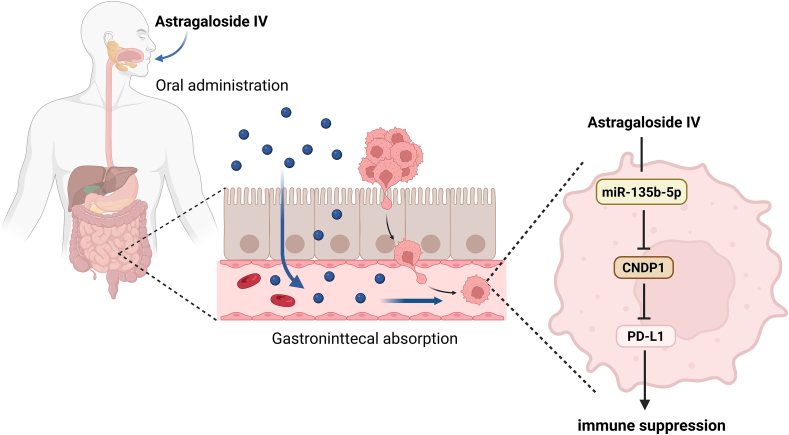
Molecular schematic representation of astragaloside IV attenuating PD-L1-mediated immune suppression through the miR-135b-5p/CNDP1 axis.

Liver cancer, particularly hepatocellular carcinoma, is the second leading cause of cancer-related death; it includes malignant tumors originating from the liver epithelial tissue. Its incidence is increasing worldwide.^56^ A previous report suggests that CNDP1 is a potential biomarker for the diagnostic and prognostic evaluation of hepatocellular carcinoma, complementing serum alpha-fetoprotein.^57^ Enhanced expression of programmed death ligand 1 (PD-L1) is closely associated with tumor cell immunosuppression. Astragaloside IV (AS-IV) reduces cell surface PD-L1 levels, thereby alleviating PD-L1-associated immune suppression through the miR-135b-5p/CNDP1 pathway.^58^ The molecular mechanism underlying this pathway is illustrated in Figure 3. CNDP1 is an independent predictor of microvascular invasion, an adverse prognostic indicator of tumor recurrence after hepatocellular carcinoma surgery.^59^

Additionally, proteomics-based clinical studies provide promising resources for discovering novel biomarkers and revealing the molecular mechanisms underlying specific diseases. Functional analysis of the datasets of hepatocellular carcinoma in a non-fibrotic liver indicated the deregulation of a protein homeostasis (proteostasis) network and the up-regulation of CNDP2. This model proposes an additional therapeutic opportunity by targeting the proteostasis network in tumors arising from a normal liver.^60^ These reports reflect the potential of CNDP1 and CNDP2 as effective biomarkers and therapeutic targets for the management of hepatocellular carcinoma.

## Gastric cancer

Gastric cancer is the fifth most common cancer and the third leading cause of cancer-related deaths worldwide. Endoscopic resection is the primary treatment for early gastric cancer, whereas advanced gastric cancer is managed with sequential lines of chemotherapy.^61^

**Figure 4:**
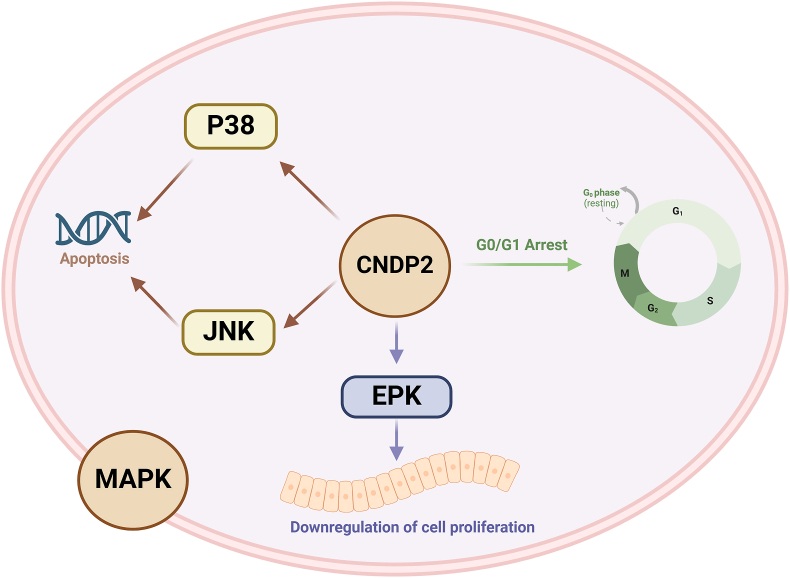
CNDP2 activates the MAPK pathway and arrests the cell cycle, acting as a functional tumor suppressor in gastric cancer.

Zhang et al have demonstrated that CNDP2 is typically down-regulated in gastric cancer tissues. The ectopic expression of CNDP2 significantly inhibited cell proliferation, induced apoptosis and cell cycle arrest, and suppressed gastric tumor growth in nude mice. Subsequent studies indicated that reintroduced CNDP2 transcriptionally up-regulated p38 and activated c-Jun NH_2_-terminal kinase (JNK), whereas the absence of CNDP2 increased the phosphorylation of extracellular signal-regulated kinase (ERK). Therefore, elevated CNDP2 levels activated the p38 and JNK–mitogen-activated protein kinase (MAPK) pathways to induce cell apoptosis, whereas its down-regulation activated the ERK–MAPK pathway to promote cell proliferation.^62^ These findings suggest CNDP2 as a tumor suppressor in gastric cancer, acting via the MAPK pathway. This mechanism is illustrated in Figure 4.

Additionally, Ying et al discovered that pepsinogen C (PGC) expression was down-regulated in gastric cancer; however, the loss of PGC could lead to resistance against chemically induced gastric cancer. PGC expression possibly inhibits the proliferation and invasion of gastric cancer cells by interacting with cyclin T1 (CCNT1), CNDP2, and cathepsin B (CTSB).^63^ These findings underscore the crucial inhibitory role of CNDP2 against gastric cancer progression.

## Renal cell carcinoma

Renal cell carcinoma (RCC) is frequently diagnosed at early stages; it is incidentally diagnosed as a small renal mass. Recently, treatment recommendations have been changed. No available biomarker can accurately predict the clinical behavior of RCC. Thus, identifying early biomarkers of RCC progression is urgently required.

Di Meo et al aimed to identify and validate early non-invasive urinary biomarkers for RCC through a quantitative label-free liquid chromatography/mass spectrometry proteomics approach combined with targeted parallel reaction monitoring and detected a significantly elevated expression of CNDP2. This outcome indicates that CNDP2 may serve as a novel biomarker for early RCC.^64^

Liquid biopsies, such as blood and urine, are significant and easily accessible sources of biomarkers and provide evidence to elucidate pathological processes. Venous-invasive RCC is a favorable model for exploring these aspects. Chinello et al used label-free nano-scale liquid chromatography-electrospray ionization-mass spectrometry to demonstrate changes in blood and urine proteomics reflecting during RCC invasion of the renal vein. The CNDP1 protein level decreases in urine with increasing vascular involvement; thus, urine potentially serves as a source of biomarkers.^65^ Overall, CNDP2 and CNDP1 are considered potential early RCC biomarkers.

## Colorectal cancer

Globally, colorectal cancer represents the fourth and third most common cancer in men and women, respectively, with its distribution significantly varying internationally.^66^ Despite genomics and proteomics-based advancements and the identification of numerous candidate biomarkers for non-invasive screening and diagnosis, more sensitive, specific, cost-effective, and patient-compliant methods are needed to effectively control this disease.

Xue et al analyzed the correlation between the clinicopathological characteristics of 183 patients and CNDP2 expression. The animal model-based experimental results indicated that CNDP2 knockdown inhibited cell proliferation through blocking cell cycle progression and delaying carcinogenesis. The CNDP2 knockdown-associated inhibition of cell proliferation and tumorigenesis involves signaling pathways, including epidermal growth factor receptor (EGFR), cyclin B1, and cyclin E. Thus, CNDP2 knockdown can inhibit colon cancer proliferation *in vitro* and delay its occurrence *in vivo*.^67^

Hua et al devised a targeted mass spectrometry method to measure the concentrations of seven plasma proteins and created a logistic regression classifier to differentiate patients with colorectal cancer from healthy subjects. They demonstrated that CNDP1 exhibits high classification accuracy for distinguishing colorectal cancer from healthy subjects, which reflected significant improvement over previously reported blood-based protein biomarkers for early colorectal cancer detection. Hence, CNDP1 can be utilized for early screening of colorectal cancer; it reflects translational potential for development into a clinically useful test.^68^ Collectively, these reports highlight the significant roles of CNDP1 and CNDP2 in the early detection and potential treatment of colorectal cancer.

## Other cancers

In ovarian cancer, CNDP2 promotes cell growth and metastasis via the phosphoinositide 3-kinase (PI3K)/protein kinase B (AKT) pathway.^69^ Quantitative proteomic analysis of bronchoalveolar lavage fluid acquired from patients with small cell lung cancer revealed elevated CNDP2 protein levels, identifying a potential marker for the small cell lung cancer subtype. Moreover, CNDP2 is a putative biomarker positively correlated with chemotherapeutic drug response; it may aid in treatment decisions for patients with small cell lung cancer.^70^ Furthermore, CNDP2 serves as a potential biomarker for the diagnosis and treatment of laryngeal cancer.^71^ Several potentially harmful variants of the CNDP2 gene were considered unlikely to account for cases of familial melanoma.^72^

CNDP1 has attracted attention owing to its regulatory effect on carnosine levels. Gautam et al (2012), for the first time, revealed that serum CNDP1 levels were reduced in patients with glioblastoma. This change may crucially influence the maintenance of carnosine levels and its bioavailability as a potential drug for treating glioblastoma.^73^ CNDP1 is considered a potential therapeutic agent for glioblastoma. In gastrointestinal cancers, lower plasma CNDP1 levels are associated with signs of catabolism and poor prognosis; hence, CNDP1 may be a hallmark of aggressive cancer and cancer cachexia.^74^ Human CNDP1 emerged as a potential biomarker of prostate cancer, with differences identified in relation to the glycosylation status of the target protein.^75^

In summary, CNDP1 and CNDP2 are variably associated with multiple cancers, including liver cancer, gastric cancer, kidney cancer, colorectal cancer, ovarian cancer, small-cell lung cancer, laryngeal cancer, pancreatic cancer, melanoma, and glioblastoma, highlighting their potential as diagnostic and therapeutic targets.

## Others

CNDP protein has many functions in addition to its role in obesity, metabolic diseases, and cancer. According to a model proposed by Bramer et al, neutrophil extracellular trap (NET) formation drives lung obstruction in COVID-19, whereas CNDP1 promotes anti-oxidant activity, pleiotropic immune responses, and vasodilation by accelerating histamine synthesis.^76^ The trinucleotide repeat (CTGs) polymorphism in *CNDP1* does not affect the survival of Chinese patients undergoing peritoneal dialysis; however, its correlation with tumor-related cardiovascular diseases needs to be validated.^77^ The reduced CNDP1 levels in patients with Parkinson's disease, multiple sclerosis, and cerebrovascular accidents are potentially attributable to hypoxic damage to carnosinase-producing cells or disruption of the blood–brain barrier.^78^ CNDP1 is an ontogeny-related gene in the liver transcriptome of children.^24^ The yeast two-hybrid analysis revealed that CNDP2 interacted with cellular proteins associated with the nonstructural protein 2 of classical swine fever virus, and suggests its potential role in virus-associated cancers.^79^ CNDP2 is a differentially expressed gene in male and female Schlager hypertensive mice, which suggests its role in tumor-related hypertension.^80^ Similarly, the differential expression of CNDP2 in lupus nephritis reflects its potential association with tumor-related autoimmune diseases.^81^

Furthermore, CNDP1 aids in the defense system by influencing antigen processing and presentation, which helps in identifying and targeting abnormal cells, such as infected or tumor cells.^82^ CNDP1/2 serves as a novel component of the antigen processing and presentation network,^82^ further highlighting its significant role in immune regulation. CNDP1 was significantly up-regulated in androgen-independent cells, which indicates the mechanism underlying its response under specific pathological conditions.^83^ The genetic relevance of CNDP1 in skin cosmetology,^84^ as well as its role in H_2_S formation,^85^ suggests its potential involvement in other physiological functions. Moreover, the monoclonal antibody RYSK173 targets the binuclear Zn center of serum CNDP1. The proportion of CNDP1 recognized by RYSK173 is inversely correlated with CNDP1 activity.^86^ *CNDP1* is a gene involved in amino acid metabolism; hence, it may play a crucial role in fructose-induced metabolic reprogramming of amino acids.^87^ High-intensity interval training increases muscle carnosine content even without the administration of dietary β-alanine^88^; this fact highlights the significance of carnosine in exercise physiology. Exposure to carnosine (CAR) up-regulates extracellular degradation via CNDP1 and induces the intracellular accumulation of CAR, suggesting a functional association between CAR and copper within the cell.^89^ Peters et al explored the association among plasma dipeptide concentrations, serum CNDP1 activity, and genotype; they detected no correlation between serum histidine dipeptide concentration and CNDP1 activity. The protective effect of low CNDP1 activity may stem from the turnover of CNDP1 substrates; alternatively, the protective function of the dipeptide might be present in other tissues.^90^ These reports demonstrate the diverse roles of CNDP in cellular metabolism and signaling.

Overall, the crucial roles of the CNDP family of proteins in disease diagnosis, metabolic regulation, cell signaling, and immune regulation are evident. A comprehensive summary of these therapeutic mechanisms is provided in Table 2. These previous reports enhance our understanding of CNDP functions and validate its effectiveness in clinical applications. However, further research can elucidate the specific mechanisms underlying various CNDP-associated physiological and pathological processes and offer new insights into the development of novel therapeutic strategies and biomarkers.

**Table 2:** Summary of therapeutic mechanisms.

Disease	CNDP1	CNDP2
Obesity	Potential therapeutic targets	Lactate+PhenylalanineCNDP2=Lac−Phe
Diabetic nephropathy	Potential biomarker	Reduce susceptibility to diabetic kidney disease
Liver cancer	miR-135b-5p/CNDP1 pathway	Potential biomarker
Gastric cancer	MAPK pathway	Potential therapeutic targets
Renal cell carcinoma	Potential biomarker	Potential biomarker
Colorectal cancer	Potential biomarker	EGFR, cyclin B1, and cyclin E
Ovarian cancer	/	PI3K/AKT pathway
Small cell lung cancer	/	Potential biomarker
Laryngeal cancer	/	Potential biomarker
Glioblastoma	Potential therapeutic agent	/
Aggressive cancer and cancer cachexia	Potential biomarker	/
Prostate cancer	Potential biomarker	/

## Outlook

This article presents an overview of the critical physiological roles of the CNDP family in various diseases, including tumors and metabolic disorders. CNDP1 and CNDP2 are significant diagnostic markers, biomarkers, and potential therapeutic targets in various cancers, including liver cancer, gastric cancer, kidney cancer, colorectal cancer, ovarian cancer, small-cell lung cancer, laryngeal cancer, pancreatic cancer, melanoma, and glioblastoma. Additionally, the versatility and clinical potential of CNDP in metabolic disorders, such as diabetic nephropathy, obesity, and cardiovascular disease, have been highlighted.

Research on CNDP in diabetes and its complications, tumors, and autoimmune diseases is expected to deepen. CNDP can potentially play a protective role in diabetic nephropathy, retinopathy, and cardiovascular complications via the regulation of CAR levels and other mechanisms. In tumors, CNDP holds great potential as an early diagnostic and therapeutic target, particularly for cancer growth and metastasis. The immunomodulatory effects of CNDP in autoimmune diseases need further investigation.

Although current research has highlighted the significant role of CNDP in various diseases, a lack of understanding of its specific molecular mechanisms, clinical application validation, and multi-disease cross-research is considerable. Further basic research and clinical trials are required to fully elucidate and validate the function of CNDP in complex pathological states and their potential applications. In conclusion, CNDP, a multifunctional protein, potentially plays crucial roles in the diagnosis and treatment of various diseases.

## CRediT authorship contribution statement

**Liang Zhou:** Methodology, Conceptualization, Writing – original draft, Data curation. **Shuxia Zhang:** Supervision. **Yunqi Zhang:** Validation. **Yun Luo:** Writing – review & editing. **Xiaobo Sun:** Writing – review & editing.

## Funding

This study was financially supported by the Key Project at central government level: The ability establishment of sustainable use for valuable 10.13039/100012573Chinese MicroResearch (No. 2060302-2305-03).

## Conflict of interests

The authors declare that the research was conducted in the absence of any commercial or financial relationships that could be construed as a potential conflict of interests.
